# Prospective qualification of early cerebral biomarkers in a randomised trial of treatment with xenon combined with moderate hypothermia after birth asphyxia

**DOI:** 10.1016/j.ebiom.2019.08.034

**Published:** 2019-08-23

**Authors:** Denis Azzopardi, Andrew T. Chew, Aniko Deierl, Angela Huertas, Nicola J. Robertson, Nora Tusor, A. David Edwards

**Affiliations:** a Centre for the Developing Brain, School of Bioengineering and Imaging Sciences, King's College London, UK; b Neonatal Department, Imperial College Healthcare NHS Trust, London, UK; c Neonatal Department, University College London Hospitals NHS Foundation Trust, London, UK; d Institute for Women's Health, University College London, UK

**Keywords:** Encephalopathy, Asphyxia, Biomarkers, Magnetic resonance spectroscopy, Magnetic resonance tensor imaging, Perinatal brain injury

## Abstract

**Background:**

The TOBY-Xe proof of concept randomised trial found no effect of xenon combined with hypothermia after birth asphyxia on the lactate to *N*-acetyl aspartate ratio (Lac/NAA) in the thalamus and fractional anisotropy (FA) in white matter tracts measured within 15 days of birth. To confirm that these biomarkers are qualified to predict long-term outcome after neural rescue therapy we assessed surviving participants at 2–3 years of age.

**Methods:**

Of the 92 infants in TOBY-Xe, one was omitted from the study, 69 survived and we assessed 62 participants, 32 in the hypothermia and xenon and 30 in the hypothermia only groups. We examined the relation between Lac/NAA and FA and the scores of the Bayley Scales of Infant and Toddler Development III and calculated their predictive accuracy for moderate or severe disability or death.

**Results:**

Fifteen of 62 participants (24%) developed moderate/severe disability, and 22/92 (24%) died.

The Lac/NAA ratio (difference in medians 0.628, 95% CI -0.392 to 4.684) and FA (difference in means −0.055, 95% CI -0.033 to −0.077) differed significantly between participants with or without moderate or severe disability or death and were significantly related with development scores in both groups. Adverse outcomes were correctly identified in 95.65% of cases by Lac/NAA and 78.79% by FA, with adequate mean calibration of the model.

**Interpretation:**

The results confirm the qualification of the cerebral magnetic resonance biomarkers employed in the TOBY-Xe study as predictors of outcome after neuroprotective therapy.

**Fund:**

The Centre for the Developing Brain, King's College London, UK.

**Research in context**

**Evidence before this study**

We searched Pubmed without language restrictions from 1st January 1990 to 1st January 2019 with the terms (xenon AND hypothermia AND outcome) and again with the terms ((magnetic resonance imaging) OR (magnetic resonance spectroscopy) AND newborns AND asphyxia AND encephalopathy).

We found just two completed randomised clinical studies of adjunct treatment with xenon and hypothermia to prevent brain injury: One was our study in newborns following birth asphyxia (The TOBY-Xe study); the other was in adults following out of hospital cardiac arrest. Both studies used magnetic resonance biomarkers for early therapeutic assessment. In the study in adults, global cerebral fractional anisotropy (FA) on magnetic resonance tensor imaging was slightly preserved in the xenon plus hypothermia group compared to the hypothermia only group, but clinical outcomes assessed six months later were similar in both groups.

Several single centre studies and one large multicentre prospective study assessed brain injury following neonatal encephalopathy by magnetic resonance spectroscopy and/or tensor imaging. In these studies, the thalamic peak area lactate/ *N*-acetyl aspartate (Lac/NAA) ratio and the NAA metabolite concentration were reported to be the optimal predictors of neurodevelopmental outcome at 2–3 years but no study has prospectively qualified these biomarkers for rapid assessment of adjunct neural rescue therapy with hypothermia following birth asphyxia.

Studies in animals using a perinatal asphyxia model assessed potential neural rescue therapies including therapies adjunct to moderate hypothermia using magnetic resonance techniques. In such studies the Lac/NAA ratio is an excellent biomarker of the therapeutic effect and consistently associated with the number of dead cells on brain immunohistochemistry and with the recovery of neurophysiological status after hypoxia ischaemia.

**Added value of this study**

The present study aimed to confirm prospectively the qualification of the thalamic Lac/NAA ratio and FA in the posterior limb of the internal capsule after an adjunct neuroprotective treatment with therapeutic hypothermia after birth asphyxia and neonatal encephalopathy. The study confirmed the high accuracy of the cerebral biomarkers chosen in the TOBY-Xe study for predicting neurodevelopmental outcome following birth asphyxia in each treatment group, and supports their use to evaluate a therapeutic effect on clinical outcomes, and estimate the power of the study to detect clinically significant changes in the cerebral biomarkers.

**Implications of all the available evidence**

Clinical trials of neuroprotective therapies initiated very soon after birth with clinical outcomes require large numbers, have a long duration and are costly. Statistically powerful qualified biomarkers measured soon after cerebral injury could enable rapid assessment of potential therapies in proof of concept studies. The present results confirm that cerebral magnetic resonance biomarkers, such as used in our study, provide a rapid and accurate surrogate outcome that can be used to identify useless interventions early and select promising neuroprotectants for further study.

Alt-text: Unlabelled Box

## 1 Introduction

Xenon, a monoatomic gas that is approved for inhalational anaesthesia, showed promising neuroprotective effects when combined with moderate hypothermia following asphyxia in studies in animals and in a preliminary clinical study in adults following cardiac arrest [[1], [2], [3], [4]]. These positive neuroprotective effects are usually attributed to inhibition of NMDA glutamate receptors and inhibition of apoptotic mechanisms [5,6].

To explore whether inhaled xenon gas combined with moderate hypothermia reduces cerebral injury in near term newborns after birth asphyxia, we carried out an open-label, proof of concept, randomised clinical trial, the TOBY-Xe study (NCT00934700), between 2012 and 2014 [7]. In that study we compared the effects of 24 h of 30% inhaled xenon combined with 72 h of hypothermia to 33.5 °C with hypothermia alone on the lactate to *N*-acetyl aspartate (Lac/NAA) peak area ratio in the thalamus measured by proton (^1^H) magnetic resonance spectroscopy (MRS) and fractional anisotropy (FA) in white matter tracts on diffusion tensor magnetic resonance imaging within 15 days of birth. These magnetic resonance measures are mechanistically relevant and clinically prognostic of severe neurological impairment. The Lac/NAA ratio measured by proton spectroscopy is a bridging biomarker which detects the effect of neuroprotective therapy in animal studies. Tract based spatial statistics with diffusion tensor imaging allows statistically powerful assessment of white matter microstructure, and the separation of small groups of subjects, thus reducing the sample size significantly compared to using visual analysis [[8], [9], [10], [11], [12], [13], [14]]. The TOBY-Xe study found no evidence of an effect of xenon combined with hypothermia on the cerebral biomarkers and on other outcomes assessed in the neonatal period. We concluded that the results did not support carrying out a large definitive clinical trial of treatment with xenon combined with hypothermia after birth asphyxia.

Cerebral magnetic resonance biomarkers have not previously been used prospectively as the primary outcomes in randomised trials of potential neuroprotective therapies following birth asphyxia and neonatal encephalopathy. Therefore, to confirm that these biomarkers are qualified to predict long-term outcome after neural rescue therapy we followed up surviving TOBY-Xe participants and performed neurodevelopmental assessment at 2–3 years of age. We hypothesized that there would be concordance between the early cerebral biomarkers and later neurodevelopmental outcome, and thus no differences on neurodevelopmental assessment between the two treatment groups.

## 2 Methods

The study was carried out in London, UK at Queen Charlotte and Chelsea Hospital, University College Hospital and the Evelina London Children's Hospital. The UK National Research Ethics Service approved the study (15/SC/0201) and the study registry identifier is NCT03968861.

The TOBY-Xe study was carried out from Jan 31, 2012, to Sept 30, 2014. We enrolled 92 infants: 46 infants randomly assigned to standard care including hypothermia to 33.5^o^ C for 72 h and 46 infants to 30% xenon for 24 h, started immediately after randomisation in the first 12 h after birth, combined with hypothermia. Infants were eligible if their gestational age was 36–43 weeks and they had at least one of the following: Apgar score of 5 or less 10 min after birth; continued need for resuscitation, including endotracheal or mask ventilation, 10 min after birth; or acidosis (defined as pH < 7 or base deficit >15 mmol/L, or both in umbilical cord blood or any blood sample) within 1 h of birth. Furthermore, eligible infants showed signs of moderate to severe encephalopathy, consisting of altered state of consciousness (reduced or absent response to stimulation), hypotonia, and abnormal primitive reflexes (weak or absent suck or Moro response), and had moderately or severely abnormal background activity for at least 30 min or seizures as shown by amplitude-integrated EEG (aEEG). We excluded infants if cooling was started after 6 h from birth or if they were older than 12 h at randomisation.

Magnetic resonance scans were carried out in TOBY-Xe participants within 15 days of birth. Details of the scanning procedures and the various sequences used in the TOBY-Xe study have been reported previously [7]. All MRS and MRI studies were done with 3·0 Tesla systems (Philips Healthcare, Best, Netherlands) at each centre. The trial research physicist undertook rigorous standardisation and a quality-control programme with phantoms and repeated scanning. Comparability test objects were transported from site to site during the project. An adult volunteer was imaged at each site periodically to provide direct comparison data. Images were obtained according to a standard protocol that included diffusion tensor MRI with 32 non-collinear directions, MRS from a single voxel on the left thalamus, and motion tolerant T1 and T2 structural scans. Tract based spatial statistics controlling for post menstrual age was used to assess diffusion tensor data for FA values. Total examination time for the study protocol was around 1 h.

Parents who consented for follow-up were invited to bring their child back for a neurodevelopmental assessment at 2 years of age. A paediatrician, trained in performing neurodevelopmental assessments and unaware of treatment allocation in the TOBY-Xe study, carried out neurodevelopmental assessments in participants at each of the three sites (AD at the Queen Charlotte and Chelsea Hospital, AH at the University College Hospital and AC at the Evelina London Children's Hospital) using the Bayley Scales of Infant and Toddler Development III [15]. AC contacted the parents of 13 children that could not be examined by their local hospital paediatrician and, with the parents' consent, carried out the assessment at the participants' homes, with their parents present.

**Fig. 1:**
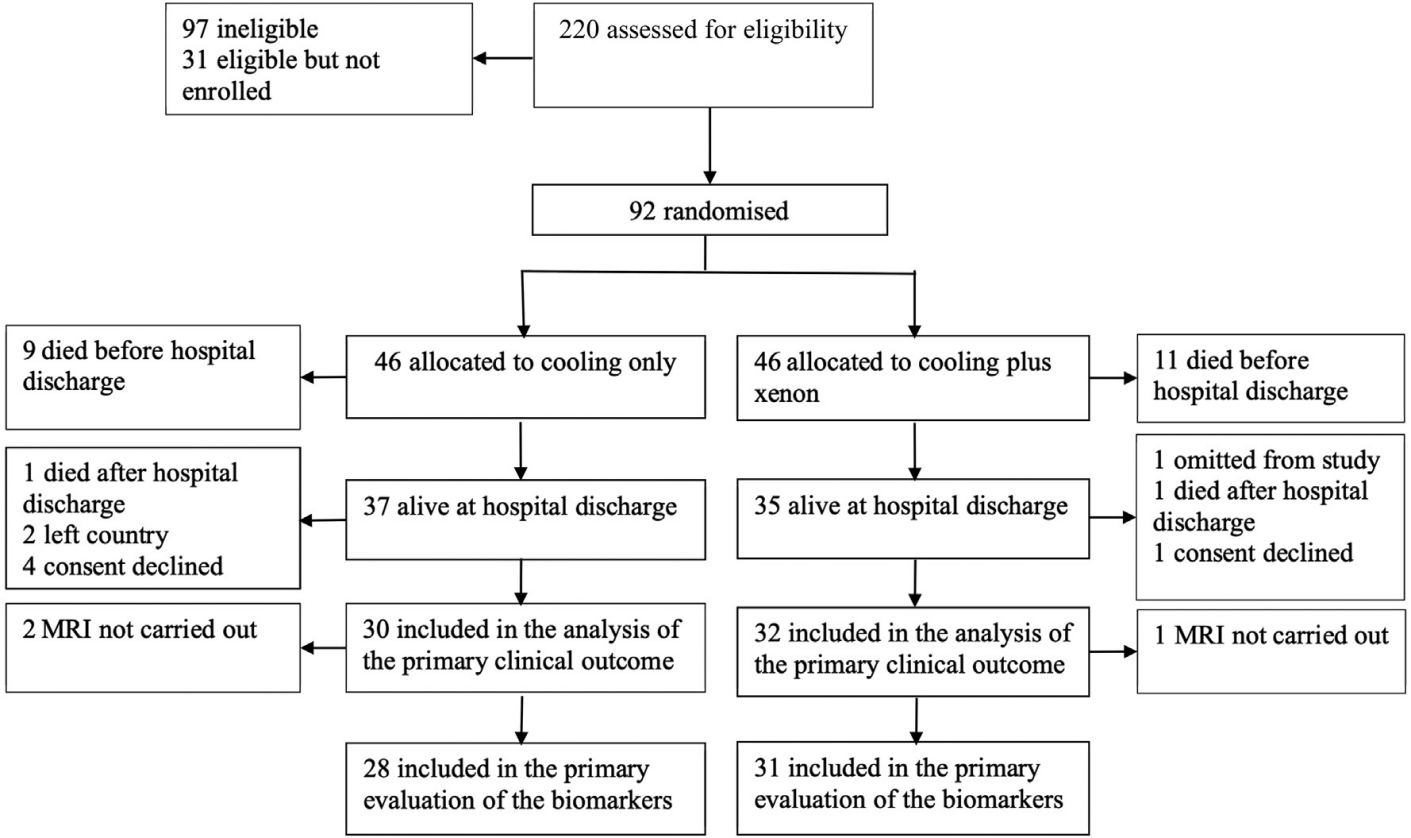
Patient flow in study.

Of the 92 newborns initially randomised, one was omitted from the original study due to incomparable magnetic resonance scanner parameters. Of the other 91 newborns recruited, 20 had died in the neonatal period and one at four months and one at seven months after birth. Two participants had left the country and 5 parents declined follow-up assessment. Finally, developmental assessments were conducted in 62 of 69 (90%) surviving children (Fig. 1 – Patient flow).

Sixty participants were assessed using the Bayley Scales of Infant and Toddler Development III and two participants older than 3.5 years were assessed using the Wechsler Preschool and Primary Scale of Intelligence, WPPSI-III [16]. Neurological assessment was by the Hammersmith Neurological Examination, reported as Optimality scores (maximum score is 78) [17]. Clinical signs of cerebral palsy were recorded and classified according to topographical distribution of the Surveillance of Cerebral Palsy in Europe classification. Functional classification was scored based on the Gross Motor Function Classification System (GMFCS) [18], while upper limb function was classified using the Manual Ability Classification System (MACS) [16]. We also collected clinical information regarding health and wellbeing since hospital discharge, including data on vision, hearing, and seizures, and medications.

### 2.1 Qualification of early cerebral biomarkers with neurodevelopmental outcomes

We examined the relation between the Lac/NAA in the thalamus measured by ^1^H MRS and FA in white matter tracts on diffusion tensor magnetic resonance and the composite scores of the Cognitive, Language and Motor scales in surviving participants, and we calculated their predictive accuracy for moderate or severe disability or death. The grade of disability was categorised as mild disability (a Cognitive score of 70 to 84, level 1 gross motor function [is able to walk independently but may have some gait abnormalities], or abnormality in one or both eyes with normal or nearly normal vision), moderate disability (a Cognitive scale score of 56 to 69, level 2 or 3 gross motor function [has minimal ability to perform gross motor skills or requires assistance with walking], or moderately reduced vision), or severe disability (a Cognitive scale score of 55 (the minimum score on the Bayley-III Cognitive scale), level 4 or 5 gross motor function [needs adaptive seating or has severely limited mobility], or no useful vision).

### 2.2 Statistical analysis

Stata (version 11) statistical software was used for the statistical analysis.

*Assessment of the relationship between early cerebral biomarkers and neurodevelopmental outcome:* Lac/NAA values were skewed so we used the natural logarithm, where appropriate. We performed linear regression to assess the relation between the Lac/NAA peak area ratio in the thalamus and FA in the posterior limb of the internal capsule to the cognitive, language and motor scale development scores, in the whole study population and by treatment subgroup. We included in the analyses the Full IQ, Verbal and Performance scores of the two children that were assessed by the WPPSI-III, as these scores have the same mean and standard deviation as those of the Bayley-III scales. We compared mean or median values of the biomarkers in the normal outcome/mild disability group and the group with moderate or severe disability or death. We analysed the receiver operating characteristic curve (ROC) and calculated the area under the curve (AUC), sensitivity, specificity, and likelihood ratios of the prediction by Lac/NAA and FA of moderate or severe disability, and repeated the analysis including deaths in the adverse outcome group. We assessed mean calibration of the prediction models by the Hosmer-Lemeshow test.

In the TOBY-Xe study, the Lac/NAA ratios were not corrected for post menstrual age at scan, therefore, we tested for a relation between these measures by linear regression.

*Analyses of clinical data of participants:* Since all continuous outcomes had non-Gaussian distributions, we present the median and interquartile range and the difference in medians with 95% confidence intervals between participants in the xenon combined with hypothermia and hypothermia only group. Wilcoxon rank-sum test was used to test the null hypothesis. We calculated relative risks and 95% confidence intervals for categorical outcomes between the two groups.

## 3 Results

The clinical characteristics at birth of the study infants are shown in Table 1, and the results of the key clinical outcome measures in Table 2. The results of the developmental and neurological assessments were similar in the the xenon/hypothermia (Xe+) and hypothermia only group (Xe-) group. Twelve of 46 participants (26.08%) from the Xe + group and 10/46 (21.74%) from the Xe- group died (Relative risk: 1.2, 95% confidence interval (CI) 0.577 to 2.498). Twenty of the 22 deaths occurred in the neonatal period and the other two deaths occurred at 4 and 7 months of age. The median (IQR) age when developmental assessment was completed was similar in both groups: Median (IQR) 25.10 (24.43 to 28.58) months in the Xe + group, and Median (IQR) 25.00 (24.29 to 29.52) months in the Xe- group (Difference in Medians, 0.10, 95% CI, 0.214 to 2.854).

**Table 1:** Baseline clinical features.

	2–3-year outcomes available			2–3-year outcomes not available
	Xenon plus hypothermia	Hypothermia only	All infants	All infants
	N = 32	N = 30	N = 62	N = 7
Treatment hospital, n (%):				
University College London	9	10	19	3
St Thomas'	12	11	23	3
Queen Charlotte and Chelsea	11	9	20	1
Birth in treatment centre, n (%):	12 (38)	10 (33)	22 (35)	1 (14)
Male sex, n (%):	19 (59)	12 (40)	31 (50)	3 (43)
Birth weight (grams):				
Mean [SD]	3417 [646]	3265 [492]	3344 [577]	3119 [406]
Gestation at delivery (weeks):				
Mean [SD]	39.9 [1.7]	39.8 [1.4]	39.8 [1.6]	40.4 [0.9]
Apgar at 10 minutes:				
Median (IQR)	5 (4 to 6.5)	5 (3 to 7)	5 (4 to 7)	4 (1 to 8)
*Unknown*	*4*	*7*	*11*	*0*
Cord/first blood pH:				
Median (IQR)	6.9 (6.8 to 7.1)	6.9 (6.8 to 7.2)	6.9 (6.8 to 7.1)	6.9 (6.7 to 7.0)
Mean [SD]	7.0 (0.2)	6.9 (0.2)	7.0 (0.2)	6.9 (0.2)
*Unknown*	*0*	*1*	*1*	*0*
Base Excess (mmol/L):				
Median (IQR)	–16.8 (–19.1 to –13.5)	–16.4 (–22.9 to –11.1)	–16.6 (–19.8 to –12.2)	–22.1 (–26.5 to –19.7)
*Unknown*	*2*	*4*	*6*	*0*
Moderate or severe encephalopathy at trial entry n (%):	32 (100)	30 (100)	62 (100)	7 (100)
Thompson hypoxic ischemic encephalopathy score at trial entry, n (%):				
0–10	5 (16)	2 (7)	7 (11)	0 (11)
11–14	19 (59)	21 (70)	40 (65)	5 (57)
15–22	8 (25)	7 (23)	15 (24)	2 (43)
Median (IQR)	13 (11 to 14.5)	13 (11 to 14)	13 (11 to 14)	14 (13 to 15)
Abnormality on aEEG at trial entry, n (%):				
Moderate	6 (19)	6 (20)	12 (19)	1 (14)
Severe	26 (81)	24 (80)	50 (81)	6 (86)
Age cooling commenced (hours), n (%):				
0 to 4 hours	27 (90)	25 (89)	52 (90)	7 (100)
4 to 6 hours	3 (10)	3 (11)	6 (10)	0 (0)
Median (IQR)	0.2 (0.1 to 2.8)	0.4 (0.1 to 0.9)	0.4 (0.1 to 1.8)	0.2 (0.1 to 3.0)
*Unknown*	*2*	*2*	*4*	*0*
Head circumference at admission to neonatal unit (cm):				
Mean [SD]	34.3 [1.4]	34.6 [1.6]	34.5 [1.5]	34.4 [1.2]
*Unknown*	*11*	*6*	*17*	*2*

**Table 2:** Neurodevelopmental outcomes at age 2–3 years.

	Xenon plus hypothermia	Hypothermia only	Difference in Medians (95% CI)	*P* value
Developmental score⁎	N = 32	N = 30		
Cognitive, Median (IQR)	90 (67.5 to 100)	90 (75 to 95)	0 (0 to 5)	*P* = .826
Language, Median (IQR)	92.5 (62 to 104.50)	91 (77 to 100)	1.5 (0 to 3)	*P* = .989
Motor, Median (IQR)	97 (64 to 103)	94 (67 to 100)	3 (0 to 8)	*P* = .874

Optimality score	*N* = 23	*N* = 22		
Median (IQR)	71 (66 to 73)	70.5 (60.50 to 76)	0.5 (2 to 0)	*P* = .794

GMFCS	N = 32	N = 30		
Level 1–2, n (%)	2 (6.25)	5 (16.67)		
Level 3–5, n (%)	6 (18.75)	3 (9.38)	RR (95% CI)^1^	1.875 (0.514 to 6.834)

MACS	N = 32	N = 30		
Level 1–2, n (%)	2 (6.25)	6 (20)		
Level 3–5, n (%)	6 (18.75)	2 (6.67)	RR (95% CI)^2^	2.813 (0.615 to 12.871)

Disability	N = 32	N = 30		
Mild, n (%)	3 (9.38)	5 (16.67)		
Moderate, n (%)	2 (6.25)	3 (9.38)	RR (95% CI)^3^	1.071 (0.443 to 2.593)
Severe, n (%)	6 (18.75)	4 (13.33)		

⁎ One child in each allocation group was tested by WPPSI-III. Their scores were: Verbal IQ 101 and 84; Performance IQ 84 and 100; Full scale IQ 90 and 89. All other participants were tested using the Bayley-III Scales.

1 Relative risk for GMFCS level 3–5.

2 Relative risk for MACS level 3–5.

3 Relative risk for moderate/severe disability.

**Fig. 2:**
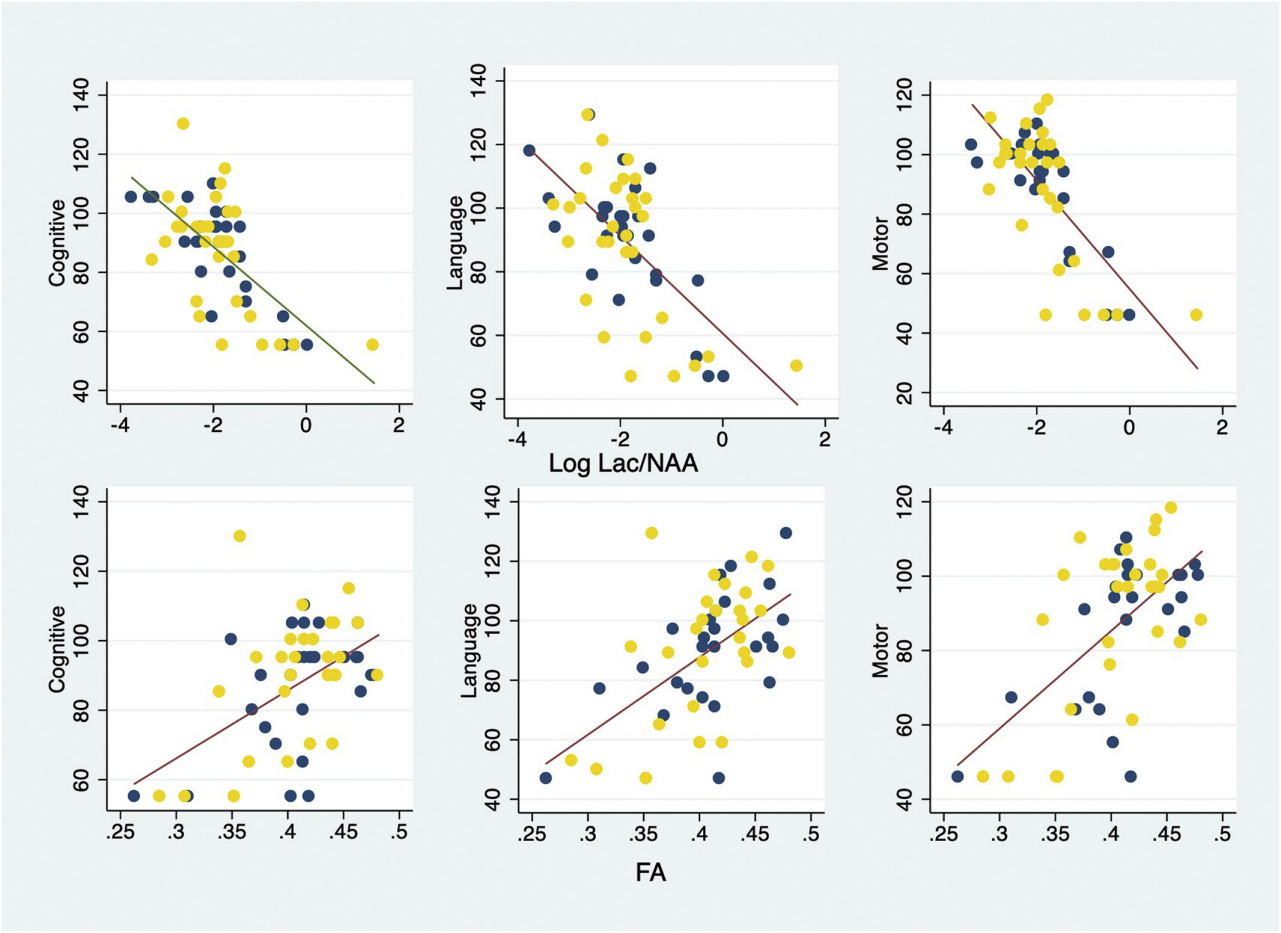
Plot of Log Lac/NAA (*N* = 57) and FA (*N* = 56) with cognitive, language and motor developmental scores.

*Relationship between early cerebral biomarkers and neurodevelopment:* The Lac/NAA ratio in the thalamus and FA in the posterior limb of the internal capsule measured within 15 days of birth were significantly related with the composite cognitive, language and motor developmental scores assessed at 2–3 years of age in the whole population and in each treatment group (Fig. 2, Table 3). The relation between the biomarkers and the developmental scale scores appeared to tail off at low values of the developmental scores, probably due to the fixed minimum scores applied in the Bayley III (Fig. 2). However, a linear function described the relationship reasonably well. Using a quadratic term in the regression did not improve the line of best fit. Excluding the data from the two infants assessed by WPPSI-III did not materially change the result of the analyses.

**Table 3:** Regression coefficients from the association of Lac/NAA ratios and FA with developmental scores.

		Coefficient (95%CI)	r^2^	P value
Cognitive				
Log Lac/NAA	Slope	−13.345 (−17.381 to −9.308)	0.444	<0.001
	Intercept	61.927 (53.636 to 70.216)		
Fractional Anisotropy	Slope	195.728 (105.935 to 285.521)	0.263	<0.001
	Intercept	7.432 (−29.420 to 44.286)		

Language				
Log Lac/NAA	Slope	−15.365 (−20.403 to −10.328)	0.400	<0.001
	Intercept	60.582 (50.238 to 70.926)		
Fractional Anisotropy	Slope	259.991 (158.404 to 361.578)	0.332	<0.001
	Intercept	−16.291 (−57.976 to 25.393)		

Motor				
Log Lac/NAA	Slope	−18.305 (−22.946 to −13.664)	0.548	<0.001
	Intercept	54.791 (45.608 to 63.976)		
Fractional Anisotropy	Slope	262.600 (167.067 to 358.133)	0.374	<0.001
	Intercept	−19.764 (−59.034 to 19.506)		

**Fig. 3:**
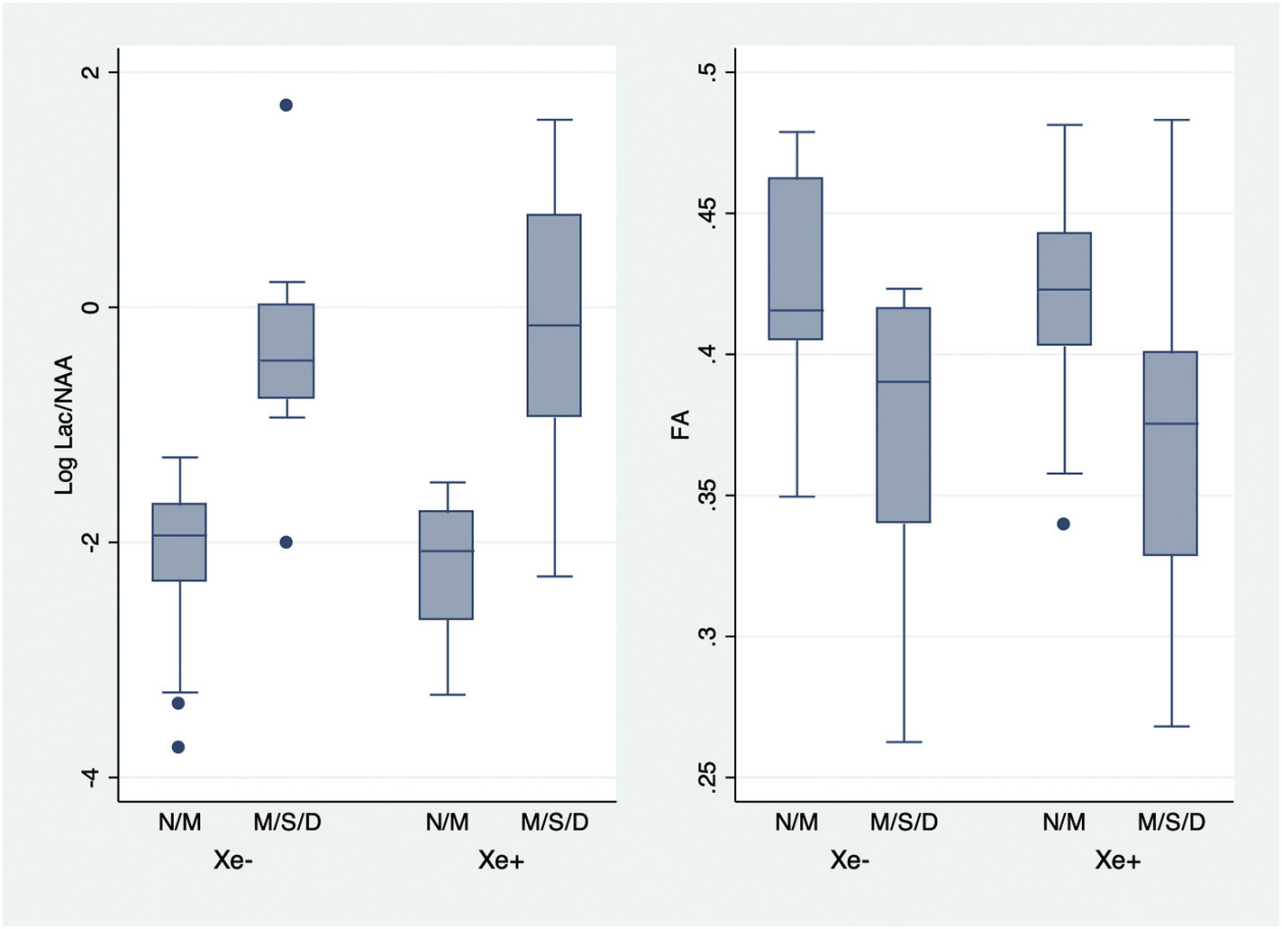
Plot of log Lac/NAA (N = 69) and FA (N = 66) values and outcome at 2–3 years of age by treatment group. Two group *t*-test of means of Lac/NAA on the log scale, in normal outcome/mild disability and moderate/severe disability/deaths groups in Xe + group, *P* < .001and Xe- groups, P < .001; Two group *t*-test of means of FA, in normal outcome/mild disability and moderate/severe disability/deaths groups in Xe+, P < .001and Xe- groups, *P* < 0.001.

**Fig. 4:**
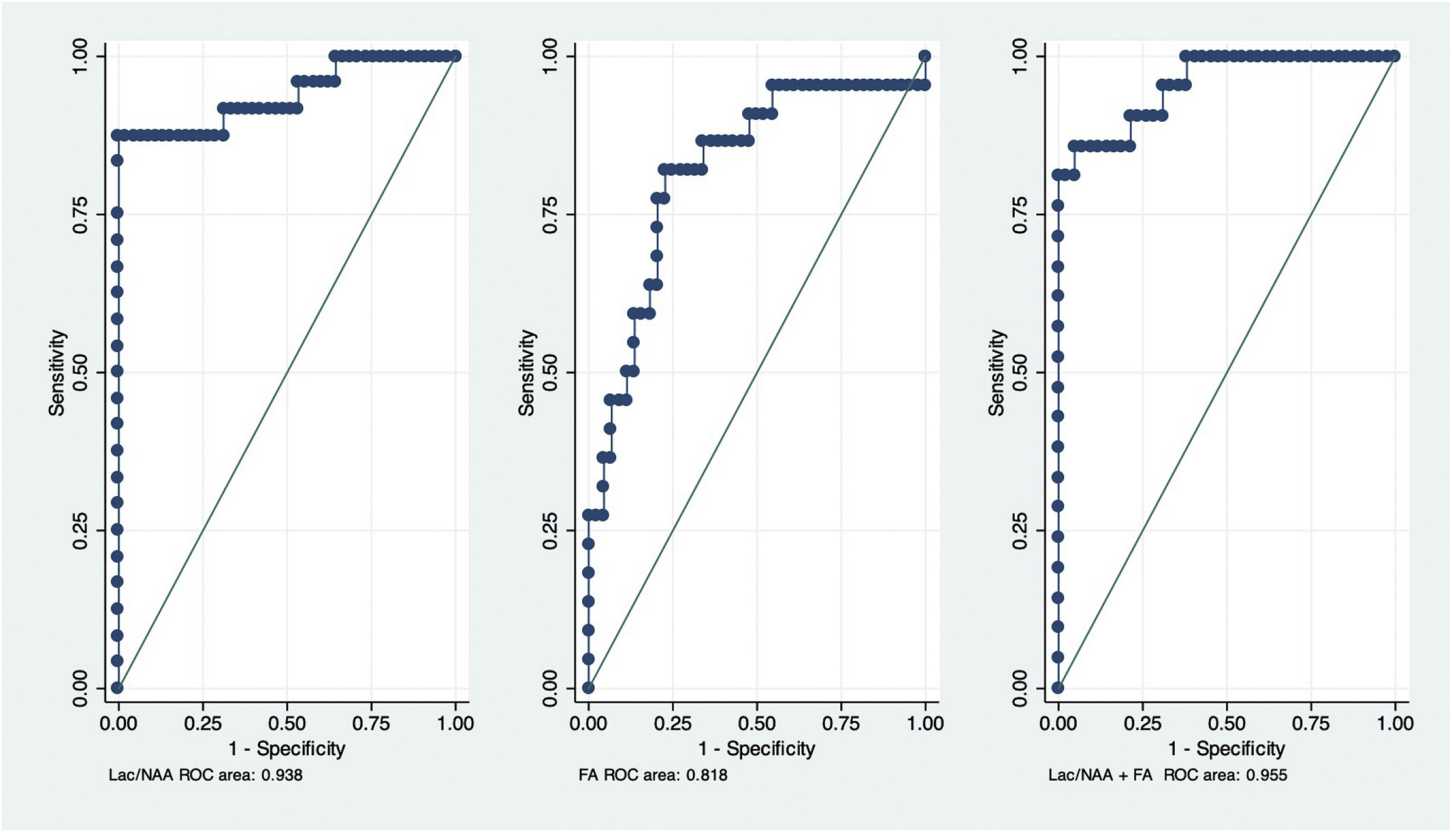
Receiver operating characteristic curves of Lac/NAA ratio (*N* = 69) and FA (*N* = 66) and the combination of Lac/NAA and FA (*N* = 63) for predicting moderate/severe disability/deaths at 2–3 years.

The Lac/NAA ratio and the FA values differed between the normal outcome/mild disability and moderate/severe disability or death groups (Fig. 3 and Table 4). Both measures also accurately predicted moderate/severe disability in the surviving participants and also when deaths were included in the adverse outcome group (Fig. 4 and Table 5). The Hosmer-Lemeshow goodness of fit test of the predictive models using all observations in the data indicated adequate mean calibration (*P* = .144 for Log Lac/NAA, *P* = .678 for FA and *P* = .483 for Lac/NAA plus FA).

**Table 4:** Biomarker values in infants with and without disability.

	Normal outcome/minor disability	Moderate/severe disability	Moderate/severe disability and deaths	Difference (95% CI)⁎	P value
Lac/NAA	*N* = 45	*N* = 12	*N* = 26		
Arithmetic mean (SD)	0.136 (0.067)	0.816 (1.13)	1.287 (1.500)	1.151 (1.59 to 0.707)	P < .001^1^
Coefficient of variation^a^	0.654	1.351	1.462		
Median [IQR]	0.142 [0.080 to 0.185]	0.600 [0.237 to 0.774]	0.770 [0.392 to 1.575]	0.628 (−0.370 to 4.684)	P = .005^2^

Fractional Anisotropy	*N* = 44	N = 12	N = 22		
Arithmetic mean (SD)	0.422 (0.034)	0.354 (0.051)	0.368 (0.055)	−0.055 (−0.033 to −0.077)	*P* < .001^3^
Coefficient of variation^b^	0.080	0.144	0.149		
Median [IQR]	0.420 [0.403 to 0.445]	0.358 [0.310 to 0.402]	0.382 [0.328 to 0.401]	−0.038 (−0.09 to −0.053)	P < .001^2^

⁎ Difference between the moderate/severe disability and deaths group and the normal outcome/minor disability group.

a This is sqrt(exp(var)-1) where var. is the variance on the log scale.

b This is the ratio of the standard deviation to the arithmetic mean in the original untransformed data.

1 Two group *t*-test of means of Lac/NAA on the log scale, in normal outcome/mild disability and moderate/severe disability/deaths groups.

2 Wilcoxon rank-sum test, in normal outcome/mild disability and moderate/severe disability/deaths groups.

3 Two group *t*-test of means of FA, in normal outcome/mild disability and moderate/severe disability/deaths groups.

**Table 5:** Predictive accuracy of early cerebral biomarkers for moderate/severe disability or death at 2–3 years.

Participating survivors at 2–3 years							
	Correctly classified (%), [cutpoint]	AUC^a^ (95% CI)	Sensitivity (%)	Specificity (%)	LR^b^+	LR^b^	P value
Lac/NAA (*N* = 57)	91.53 [≥0.308]	0.752 (0.548 to 0.954)	64.29	100.00	Infinite	0.357	0.001
FA (N = 56)	87.50 [≤0.363]	0.877 (0.773 to 0.982)	66.67	93.18	9.777	0.357	0.001
Lac/NAA + FA (*N* = 52)	94.20 [≥0.591]	0.914 (0.807 to 1.000)	70.00	100.00	infinite	0.300	0.002

Participants including deaths							
Lac/NAA (*N* = 71)	95.65 [≥0.308]	0.938 (0.866 to 1.000)	88.46	100.00	infinite	0.153	<0.001
FA (N = 66)	78.79 [≤0.403]	0.818 (0.703 to 0.934)	81.82	77.27	3.600	0.253	<0.001
Lac/NAA + FA (N = 63)	93.65 [≥0.611]	0.955 (0.903 to 1.000)	80.95	95.24	17.006	0.200	<0.001

a AUC: area under the curve.

b LR: Likelihood ratio.

**Fig. 5:**
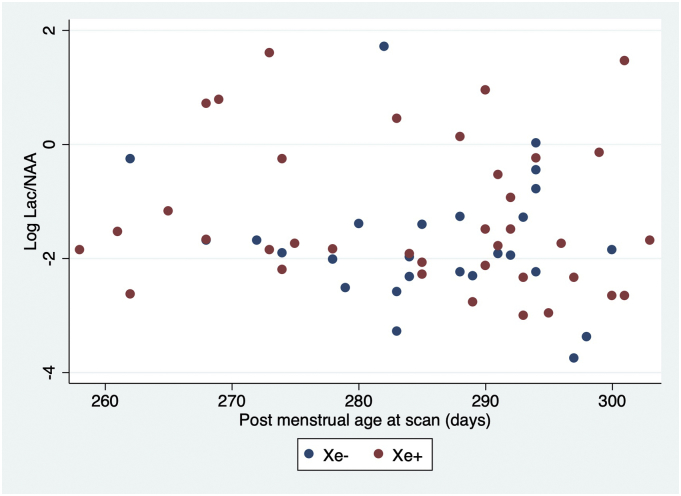
Plot of Log Lac/NAA and post menstrual age in days at scan (N = 63).

There was no correlation between the Lac/NAA ratios and post menstrual age at scan (*P* = .272, r^2^ 0.020, Fig. 5).

## 4 Discussion

The Lac/NAA ratio in the thalamus and FA in the posterior limb of the internal capsule measured within 15 days of birth were highly correlated with neurodevelopmental outcomes and accurately predicted the occurrence of moderate or severe disability in the surviving children or death in each randomised group. The results confirm the qualification of the cerebral magnetic resonance biomarkers employed in the TOBY-Xe study as predictors of outcome after neuroprotective therapy following birth asphyxia.

Recent studies confirmed the accuracy of MRS and MRI biomarkers for predicting an adverse outcome following birth asphyxia and neonatal encephalopathy, but without testing any therapeutic intervention.(10;11) The present study thus aimed to confirm prospectively the qualification of the cerebral biomarkers after an adjunct neuroprotective treatment with therapeutic hypothermia. Studies in animals have shown that the thalamic Lac/NAA ratio is a sensitive biomarker of the therapeutic and biological effects of hypothermia and of adjunct treatment with hypothermia following asphyxia, and we have previously shown the value of MR imaging as a biomarker after therapeutic hypothermia in a retrospective analysis of the TOBY randomised trial of therapeutic hypothermia [[19], [20], [21], [22]]. In such studies, using MRS sequences at 3 T similar to those in babies with neonatal encephalopathy, Lac/NAA is consistently associated with the number of dead cells on brain immunohistochemistry and with the recovery of aEEG after hypoxia ischaemia. Translating these pre-clinical data to the clinical TOBY-Xe study gives us confidence in Lac/NAA to detect a biological effect or not as is the case here with the addition of xenon to hypothermia. Such studies show that the Lac/NAA ratio is robust enough to clearly demonstrate a biological effect if it existed. The TOBY-Xe study failed to show a therapeutic effect of adjunct treatment with xenon in neonatal encephalopathy, and our results confirm that the cerebral biomarkers do not detect an effect that is not real. Our results demonstrate the high predictive accuracy of the cerebral biomarkers in both randomised groups. Our data, together with the data from pre-clinical and clinical observational studies show that the cerebral biomarkers chosen in the TOBY-Xe study are accurate in predicting later outcomes following neural rescue therapy, supporting their use to assess a therapeutic effect on clinical outcomes in the two outcome groups, and estimate the power of the study to detect clinically significant changes in the cerebral biomarkers.

The MARBLE study reported that the NAA metabolite concentration in the thalamus was the optimal predictor following neonatal encephalopathy, but another recent study reported a similar predictive accuracy for the thalamic Lac/NAA ratio [11]. The predictive performance of the Lac/NAA ratio in our study was nearly identical to that reported in the MARBLE study when deaths are included in the adverse outcome group (AUC 0.94, 95% CI 0.866–1.000 in MARBLE and AUC 0.938, 95% CI 0.866–1.000 in this study). The data are thus consistent. In the MARBLE study thalamic metabolite concentrations were obtained in 43% of participants, and peak area thalamic metabolite ratios in 84% of infants. Our multicenter study using purposely developed scanning protocols and compatible scanners acquired peak area thalamic metabolite ratios from all 78 infants who had an MRI scan in the neonatal period and FA data from 73/78 (95%) infants, proving the feasibility of using these biomarkers in clinical trials.

Although standard magnetic resonance imaging with visual assessment is now commonly used to assess the severity of cerebral injury and guide management following birth asphyxia and neonatal encephalopathy, the high accuracy for predicting later neuro-developmental outcome and the feasibility of measuring the Lac/NAA ratio on ^1^H MRS confirmed in this study indicate that this is potentially a clinically useful measure that could be incorporated into clinical practice. However, since ^1^H MRS studies in newborns have been in specialist centres, further clinical studies are needed to determine the feasibility and cost/benefits of introducing this measure routinely in infants with neonatal encephalopathy.

Two infants, one from each allocation group, had zero Lac/NAA values on proton MRS, and both infants developed severe disabilities. Absence of a lactate signal following severe asphyxial encephalopathy is highly unusual and we cannot exclude that the infants had an alternative pathology. These two data points are dropped following log normal transformation as log normal transformation of zero values is not possible.

Studies in animals have suggested that there are several potential neuroprotectants that might augment the modest therapeutic effect of moderate hypothermia following birth asphyxia. However, clinical trials of adjunct neuroprotective therapies initiated very soon after birth with clinical outcomes require large numbers, have a long duration and are costly. There is a critical need for statistically powerful qualified biomarkers measured soon after cerebral injury for rapid assessment of potential therapies in proof of concept studies. The present results confirm that cerebral magnetic resonance biomarkers, such as used in our study, provide a rapid and accurate surrogate outcome that can be used to identify useless interventions early and select promising neuroprotectants for further study.

Neurological and neurodevelopmental outcomes at 2–3 years of age were very similar in the xenon plus hypothermia and hypothermia only groups, mirroring the closeness of the results between groups of the cerebral biomarkers measured soon after birth, visual analysis of conventional MRI and neurological assessment at hospital discharge in the neonatal period. There was no difference in deaths and loss to follow-up in the two randomised groups, so the randomisation property still broadly applies to the 2–3-year outcomes, but this study was not designed to detect an effect of xenon on neurodevelopmental outcomes and was underpowered to do so because TOBY-Xe was powered to detect changes in the cerebral biomarkers. However, the near identical results of the clinical outcomes in the two treatment groups, and the close concordance with the neonatal outcomes suggest a low probability that the results would materially change with a larger study.

The results also confirm that a definitive clinical trial of xenon gas combined with moderate hypothermia after birth asphyxia would not be appropriate using the protocol defined in the TobyXe study. The absence of a beneficial effect of xenon could be for a number of reasons, such as: suboptimal dose, duration and timing of administration of xenon (the mean age of starting xenon in TOBY-Xe was almost 10 h); the severity of encephalopathy in the participants; low sensitivity of the cerebral biomarkers used in the study; and low power of the follow-up study. Neuroprotective therapies are less likely to be successful following more severe injury, as was demonstrated in a recent study in animals that found no cerebral protection with xenon following a severe asphyxial insult whilst previous studies found beneficial effects following milder injury [23]. However, it is unlikely that the TobyXe study participants were more severely affected than infants recruited to the successful large randomised trials of cooling since the eligibility criteria of the TobyXe study were similar to those of the cooling trials, and the mortality and severe disability rates did not differ greatly from that reported in those trials [24].

## Funding sources

The UK Medical Research Council funded the TOBY-Xe study, and participating hospitals and The Centre for the Developing Brain, King's College London, UK, supported the follow-up study.

The funder of the TOBY-Xe study had no role in study design; data collection, analysis, or interpretation; or writing of the report. The corresponding author had full access to all the data in the study and the final decision to submit for publication was made by the authors. All authors approved the final version of the manuscript submitted for publication.

## Author contributions

Prof D Azzopardi and Prof AD Edwards originated and designed the study, analysed the data and wrote the first draft of the manuscript. Prof NJ Robertson, Dr. AT Chew, Dr. A Huertas, Dr. A Deierl, and Dr. N Tusor contributed to the study design, data analysis and data collection. All authors contributed to the writing of the final version of the manuscript.

## Declaration of Competing Interest

The authors report no financial interest of potential conflict of interests

## References

[1] Liu X., Dingley J., Scull-Brown E., Thoresen M. (2015). Adding 5 h delayed xenon to delayed hypothermia treatment improves long-term function in neonatal rats surviving to adulthood. Pediatr.Res..

[2] Ma D., Hossain M., Chow A., Arshad M., Battson R.M., Sanders R.D. (2005). Xenon and hypothermia combine to provide neuroprotection from neonatal asphyxia. Ann.Neurol..

[3] Laitio R., Hynninen M., Arola O., Virtanen S., Parkkola R., Saunavaara J. (2016). Effect of inhaled xenon on cerebral white matter damage in comatose survivors of out-of-hospital cardiac arrest: a randomized clinical trial. JAMA.

[4] Faulkner S., Bainbridge A., Kato T., Chandrasekaran M., Kapetanakis A.B., Hristova M. (2011). Xenon augmented hypothermia reduces early lactate/N-acetylaspartate and cell death in perinatal asphyxia. Ann.Neurol..

[5] Harris K., Armstrong S.P., Campos-Pires R., Kiru L., Franks N.P., Dickinson R. (2013). Neuroprotection against traumatic brain injury by xenon, but not argon, is mediated by inhibition at the N-methyl-D-aspartate receptor glycine site. Anesthesiology.

[6] Ma D., Williamson P., Januszewski A., Nogaro M.C., Hossain M., Ong L.P. (2007). Xenon mitigates isoflurane-induced neuronal apoptosis in the developing rodent brain. Anesthesiology.

[7] Azzopardi D., Robertson N.J., Bainbridge A., Cady E., Charles-Edwards G., Deierl A. (2016). Moderate hypothermia within 6 h of birth plus inhaled xenon versus moderate hypothermia alone after birth asphyxia (TOBY-Xe): a proof-of-concept, open-label, randomised controlled trial. Lancet Neurol.

[8] Thayyil S., Chandrasekaran M., Taylor A., Bainbridge A., Cady E.B., Chong W.K. (2010). Cerebral magnetic resonance biomarkers in neonatal encephalopathy: a meta-analysis. Pediatrics.

[9] Tusor N., Wusthoff C., Smee N., Merchant N., Arichi T., Allsop J.M. (2012). Prediction of neurodevelopmental outcome after hypoxic-ischemic encephalopathy treated with hypothermia by diffusion tensor imaging analyzed using tract-based spatial statistics. Pediatr.Res..

[10] Lally P.J., Montaldo P., Oliveira V., Soe A., Swamy R., Bassett P. (2019). Magnetic resonance spectroscopy assessment of brain injury after moderate hypothermia in neonatal encephalopathy: a prospective multicentre cohort study. Lancet Neurol.

[11] Mitra S., Kendall G.S., Bainbridge A., Sokolska M., Dinan M., Uria-Avellanal C. (2019). Proton magnetic resonance spectroscopy lactate/N-acetylaspartate within 2 weeks of birth accurately predicts 2-year motor, cognitive and language outcomes in neonatal encephalopathy after therapeutic hypothermia. Arch Dis Child Fetal Neonatal Ed.

[12] Berger H.R., Brekke E., Wideroe M., Morken T.S. (2017). Neuroprotective treatments after perinatal hypoxic-ischemic brain injury evaluated with magnetic resonance spectroscopy. Dev Neurosci.

[13] Groenendaal F., de Vries L.S. (2017). Fifty years of brain imaging in neonatal encephalopathy following perinatal asphyxia. Pediatr Res.

[14] Robertson N.J., Thayyil S., Cady E.B., Raivich G. (2014). Magnetic resonance spectroscopy biomarkers in term perinatal asphyxial encephalopathy: from neuropathological correlates to future clinical applications. Curr Pediatr Rev.

[15] Bayley N. (2006). Bayley scales of infant and toddler development: Administration manual.

[16] Wechsler D. (2003). Wechsler Preschool & Primary Scale of Intelligence - Third UK Edition (WPPSI-III UK).

[17] Haataja L., Mercuri E., Guzzetta A., Rutherford M., Counsell S., Flavia F.M. (2001). Neurologic examination in infants with hypoxic-ischemic encephalopathy at age 9 to 14 months: use of optimality scores and correlation with magnetic resonance imaging findings. J Pediatr.

[18] Palisano R.J., Rosenbaum P., Bartlett D., Livingston M.H. (2008). Content validity of the expanded and revised gross motor function classification system. Dev Med Child Neurol.

[19] Amess P.N., Penrice J., Cady E.B., Lorek A., Wylezinska M., Cooper C.E. (1997). Mild hypothermia after severe transient hypoxia-ischemia reduces the delayed rise in cerebral lactate in the newborn piglet. Pediatr Res.

[20] Robertson N.J., Faulkner S., Fleiss B., Bainbridge A., Andorka C., Price D. (2013). Melatonin augments hypothermic neuroprotection in a perinatal asphyxia model. Brain.

[21] Broad K.D., Fierens I., Fleiss B., Rocha-Ferreira E., Ezzati M., Hassell J. (2016). Inhaled 45-50% argon augments hypothermic brain protection in a piglet model of perinatal asphyxia. Neurobiol Dis.

[22] Rutherford M., Ramenghi L.A., Edwards A.D., Brocklehurst P., Halliday H., Levene M. (2010). Assessment of brain tissue injury after moderate hypothermia in neonates with hypoxic-ischaemic encephalopathy: a nested substudy of a randomised controlled trial. Lancet Neurol.

[23] Sabir H., Osredkar D., Maes E., Wood T., Thoresen M. (2016). Xenon combined with therapeutic hypothermia is not neuroprotective after severe hypoxia-ischemia in neonatal rats. PLoS One.

[24] Jacobs S.E., Berg M., Hunt R., Tarnow-Mordi W.O., Inder T.E., Davis P.G. (2013). Cooling for newborns with hypoxic ischaemic encephalopathy. Cochrane Database Syst Rev.

